# 97. Pharmacist Driven Antimicrobial Stewardship Interventions on the Management of Urinary Tract Infections in the Emergency Department at a Tertiary Military Treatment Facility

**DOI:** 10.1093/ofid/ofab466.299

**Published:** 2021-12-04

**Authors:** Memar D Ayalew, Sorana raiciulescu, Daniel Brooks, Robin Williams, Paulette Crull, Roseanne Ressner

**Affiliations:** 1 Walter Reed National Military Medical Center, Springfield, Virginia; 2 Uniformed Services University Biostatistics Consulting Center, Bethesda, Maryland; 3 WRNMMC, Bethesda, Maryland; 4 Walter Reed Military Medical Center, Rockville, Maryland; 5 Walter Reed National Military Medical Center, Bethesda, MD, Bethesda, Maryland

## Abstract

**Background:**

With the Joint Commission standards targeting ambulatory settings serving as a catalyst, we designed a quality improvement (QI) project was designed to evaluate the existing management and prescribing patterns for urinary tract infections (UTI) in the Walter Reed National Military Medical Center (WRNMMC) Emergency Department (ED) in order to identify targets for ASP intervention.

**Methods:**

This was a Pharmacist-driven, prospective, QI project conducted over a 3-month period. The clinical presentations and microbiological data of uncomplicated cystitis and pyelonephritis cases managed in the ED were reviewed. Within 24-72 hours of ED discharge, recommendations were relayed to both patients and ED staff. Diagnostic criteria and management concordant with established clinical guidelines were assessed. Inclusion criteria included age ≥ 18, admission status, urine culture and antibiotics for UTI or pyelonephritis.

**Results:**

A daily urinalysis (UA) report identified 1781 ED encounters of which 117 cases met inclusion criteria. Nitrofurantoin was most prescribed empirically at 39.3% followed by a cephalosporin (23.1%) or a fluoroquinolone (19.7%), accounting for 32% of inappropriate empiric antibiotic selection. Cases were identified with inappropriate duration of therapy (22.2%), dosage (9.4%), and drug-bug mismatch (9.4%). Nearly 38% of cases required intervention to discontinue (32.5%) or initiate new antibiotics (3.4%). Diagnostic concordance was defined as having positive urinary symptoms, clinically significant UA and positive urine culture. This was only observed in 37.6% of all cases, of which only 43.2% were treated with a guideline concordant empiric regimen, dosage and duration of therapy. Although not included in the final analysis, it was noted 916 urine culture results were ordered where 70% were not associated with genitourinary complaints or sepsis.

**Conclusion:**

Despite guidelines for UTI management, considerable practice discordance was found in the ED. Multiple Pharmacist targeted interventions were identified. Prioritized areas for ED provider education include first-line therapy, treatment duration, and diagnostic stewardship. This QI project has potential for optimizing prescribing practices in Military Health System ambulatory settings.

**Disclosures:**

**All Authors**: No reported disclosures

